# Using Dynamic Stochastic Modelling to Estimate Population Risk Factors in Infectious Disease: The Example of FIV in 15 Cat Populations

**DOI:** 10.1371/journal.pone.0007377

**Published:** 2009-10-16

**Authors:** David Fouchet, Guillaume Leblanc, Frank Sauvage, Micheline Guiserix, Hervé Poulet, Dominique Pontier

**Affiliations:** 1 Université de Lyon, Lyon, France; 2 Université Lyon 1, CNRS, UMR5558, Laboratoire de Biométrie et Biologie Evolutive, Villeurbanne, France; 3 Merial, Recherche et Développement, Lyon, France; University of Oxford, United Kingdom

## Abstract

**Background:**

In natural cat populations, Feline Immunodeficiency Virus (FIV) is transmitted through bites between individuals. Factors such as the density of cats within the population or the sex-ratio can have potentially strong effects on the frequency of fight between individuals and hence appear as important population risk factors for FIV.

**Methodology/Principal Findings:**

To study such population risk factors, we present data on FIV prevalence in 15 cat populations in northeastern France. We investigate five key social factors of cat populations; the density of cats, the sex-ratio, the number of males and the mean age of males and females within the population. We overcome the problem of dependence in the infective status data using sexually-structured dynamic stochastic models. Only the age of males and females had an effect (*p* = 0.043 and *p* = 0.02, respectively) on the male-to-female transmission rate. Due to multiple tests, it is even likely that these effects are, in reality, not significant. Finally we show that, in our study area, the data can be explained by a very simple model that does not invoke any risk factor.

**Conclusion:**

Our conclusion is that, in host-parasite systems in general, fluctuations due to stochasticity in the transmission process are naturally very large and may alone explain a larger part of the variability in observed disease prevalence between populations than previously expected. Finally, we determined confidence intervals for the simple model parameters that can be used to further aid in management of the disease.

## Introduction

Feline Immunodeficiency Virus (FIV) infects numerous feline species worldwide [1]. This *Lentivirus* from *Retroviridae* family is closely related to Human Immunodeficiency Virus (HIV) and Simian Immunodeficiency Virus (SIV) [2]. This is a virus of major importance because it is lethal to the domestic cat (*Felis silvestris catus*) and can affect several other cat species, most of which are threatened or endangered e.g., the European wildcat *F. s. silvestris* in Europe [3]–[5]. There is thus a need to better understand the risk factors affecting the spread and patterns of persistence of FIV in natural populations of domestic cats.

In natural domestic cat populations, FIV is mainly transmitted through bites arising from aggressive or sexual contacts [3], [6]–[10]. As a consequence, the spread of FIV in domestic cat populations is highly influenced by the mating system; a higher FIV prevalence is observed in aggressive and polygynous cat populations that involve more fights and bites than in much less aggressive and promiscuous urban ones [8], [9], where FIV can be absent [11].

Basically, factors affecting cats’aggressiveness can be divided into two categories. At the **individual** level, some cats are more aggressive than others. Typically, this is the case for dominant males [8], [9] or orange cats [12]. In the field, they are generally more often infected than subordinates, females or other colour morphs [8], [12], [13]. At the **population** level, the overall aggressiveness of cats largely depends on the population social structure. A male-biased sex-ratio may make the entire population more aggressive, making virus transmission more efficient and, thus, lead to higher disease prevalence.

Until now, to our knowledge, all studies of FIV risk factors have focused on individual risk factors. Factors that may increase the overall virus transmission rate are at least as important for controlling the disease spread but, paradoxically, have been largely overlooked until now. Here, we investigate how some characteristics of cat populations, such as cat density or sex-ratio, e.g., as indicators for aggressiveness in contacts within the population, may act as population risk factors that increase or decrease the virus prevalence within populations.

Understanding the factors that may increase the FIV transmission rate within populations requires the sampling of a set of neighboring cat populations (which, until now, has rarely been done), and then examination of how FIV prevalence correlates with the suspected risk factors. For that purpose, we sampled 15 cat populations in North-Eastern France and measured, within each population, FIV prevalence in males and females. We found significant variability in disease prevalence between populations, especially in males. We also measured five social indicators in order to measure how they correlated with FIV prevalence.

Commonly, risk factors are analyzed with logistic regression models. However, these models are built on the assumption that individuals become infected independently of each other; a hypothesis that contradicts the fundamental communicable nature of infectious diseases [1], [14], [15]. Moreover, as described below, assumptions of independence lead to underestimate the variability in disease prevalence between populations that would be observed in the absence of risk factors.

Our method is inspired by previous works based on the comparison of stochastic dynamic models of the disease spread within host populations to the data [14], [16]–[20]. The idea is that each combination of population risk factors leads to a different model. Our objective is to determine the model (i.e. the combination of risk factors) that best fits the data. Beyond the simple analysis of the risk factors associated with FIV, this work aims to understand why the variability observed in our disease prevalence data is so large - data on disease prevalence in males exhibited significant extra-Binomial variations. Can we isolate population risk factors that would explain particularly high disease prevalence in some populations? Does the spatial aggregation of populations with high virus prevalence help to explain the variability in disease prevalence? Or, in contrast, is the large variability observed in disease prevalence a natural consequence of the transmissible nature of the virus?

The work presented here supports this last hypothesis: random fluctuations in the transmission process lead to much greater variation in disease prevalence than with a simple Binomial distribution, underlying classical risk factor analyses. So the simplest model describes well the data and explains the large variability observed in disease prevalence between cat populations without invoking any risk factor. Finally, we determine confidence intervals for the model parameters. The model is very simple, explains the data well, and hence constitutes an interesting tool for further understanding and control of the spread of FIV in these cat populations. The approach developed here can easily extend to many host-parasite interactions.

## Materials and Methods

### 1. Data set

The field work has been made by qualified people according to current French legislation. Accreditation has been granted to the UMR-CNRS 5558 (accreditation number 692660703) for the program.

**Figure 1:**
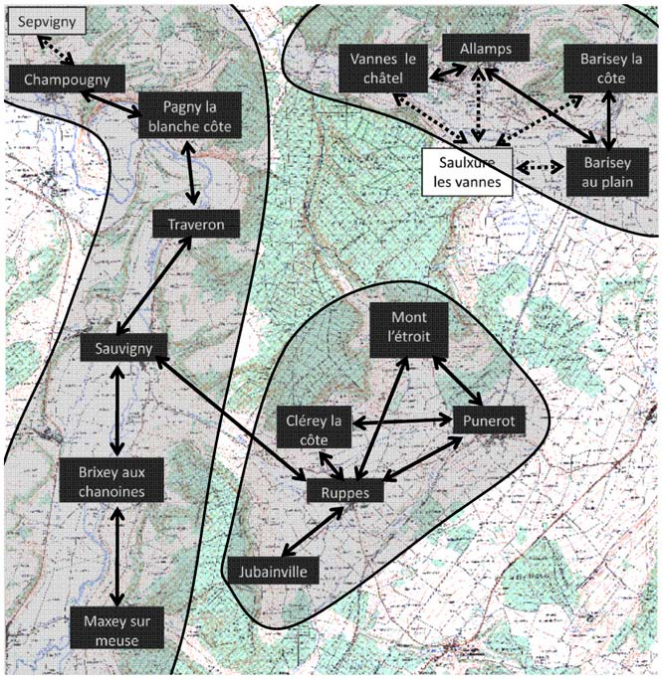
The study area. We identified three metapopulations (grey areas). Studied cat populations are represented with black rectangles and solid arrows represent the suspected interactions between the studied populations. Some unstudied populations may interact with the studied ones (dashed arrows) and are represented by white rectangles.

Fifteen spatially separated rural cat populations were sampled during 2007 in North-Eastern France near the city of Nancy (Fig. 1, black rectangles). The distance separating neighboring cat populations varied from 1.2 to 4 km. The study zone covers a territory of approximately 250 km^2^. In order to delimit the study area, we considered the geographical characteristics that might limit movements between the studied cat populations and those outside of the studied area. The spatial organisation of geographical barriers suggests that the populations may be organized into three distinct metapopulations, with rare contacts between cats of different metapopulations (Fig. 1, grey areas). At a finer scale, behavioral observations reveal that males can disperse between populations along roads. By adopting a basic assumption that populations are considered connected when they are not too distant from each other (i.e. less than 2 km) and are connected by roads, we propose a connection network between the different populations (see Fig. 1, solid arrows).

Unfortunately, it was not possible to establish a fully isolated perimeter (at least in relation to spread of FIV), so some populations of the study area are in fact connected to unstudied populations (Fig. 1, white rectangles - the connections to the study populations are represented by dashed arrows). In particular, Saulxure-Les-Vannes is connected to several study populations. However, it was not considered for sampling because of potential bias due to a cat culling programme there. The village of Sepvigny, which is connected to Champougny, could not be sampled for technical reasons.

Most of the cats were captured using baited traps; others being caught directly in their owner’s houses. Upon capture, cats were anaesthetized with an intramuscular injection of ketamin chlorhydrate (Immalgène 1000 15 mg/kg, Rhône-Mérieux) and acepromazin (Vétranquil 5.5% 0.5 mg/kg, Sanofi). They were marked permanently using an electronic passive integrative transponder (pig-tag) to allow all individuals to be identified in case of recapture. For each cat, we have recorded, among other data, information on sex, age and serological status in relation to FIV. Blood samples were taken from the jugular vein and the cats were then released. The ELISA method (SNAP Combo +, Idexx) was used to detect the presence of FIV-specific antibodies, which generally identifies virus carriers [6]. All positive sera for FIV were confirmed by Western blot analysis [21]. FIV was scored as present or absent for each sampled cat.

### 2. Statistical analysis

#### 2.1 General approach

The approach we use here is very similar to the classical approach based on multifactorial logistic regression, which consists of:

- *Step 1: Choice of a model H^0^ against which the data is compared*. In the case of the classical logistic regression approach, it is assumed that all individuals have a same probability *p* to be infected, **independently of the other individuals’ status**. As a result, under model *H^0^* the distribution of the number of infected cases in the population follows a binomial distribution of parameter *p* and *N*, where *N* is the number of individuals of the population.
- *Step 2: Some of the model parameters are expected to depend on risk factors.* Choosing *p* as a function of risk factors means that each individual has its own probability of being infected (depending on its characteristics in terms of risk factors). It is classically assumed that the logit of *p* is a linear function of the different risk factors: *logit(p) = a_0_+∑a_i_X_i_*, where *X_i_* denotes the *i*-th risk factor value for the individual.
- *Step 3: Model selection process.* Different models are defined by setting some coefficients (*a_i_*) to 0. Hence, the probability of being infected only depends on the risk factors which associated coefficients are non-zero. The different models are compared (usually using an Akaïke Information Criterium, AIC) to determine which model best describes the data.

Note that there are two equivalent ways of presenting the classical approach. Firstly, the expected **proportion** of infected captured individuals is taken as a function of risk factors plus a random term based on a centered binomial distribution. Secondly, the **probability** that each captured individual is infected is taken as a function of risk factors, with random fluctuations in expected proportions naturally arising from these probabilities. Here, we present the second format because it allows us to easily illustrate how our approach is, in fact, a natural extension of the classical one.

The main difference between our approach and the classical one comes from the model used to describe the data. It is quite obvious that for transmissible diseases the probability of one individual being infected is not independent of the infection status of the other individuals [1], [14], [15]. Here we consider the probability of individuals becoming infected as the result of a dynamic process of between-host virus transmission (described in the next section). These types of models are widely recognized as common tools for representing infectious disease data.

We also make some minor changes to steps 2 and 3. In step 2, the logit function is chosen in the classical approach mainly because the model parameter *p* is bounded by 0 and 1. Since, as described below, our model parameters are not bounded by 1, we have no reason to consider their logit value. Lastly, in step 3 for model comparison we use likelihood ratio tests (LRT) rather than AIC. LRTs are chosen to test one particular assumption, which is here whether the simplest model, *i.e.* where no model parameter depends on risk factors, is sufficient to describe the data.

#### 2.2. The dynamic epidemiological model – Model *H^0^*

The aim of this framework is to study population risk factors, *i.e*. factors that affect the rate at which the virus is transmitted within the population. Individual risk factors, *i.e*. factors that make some individuals more prone to infection than others in the same population, are not studied here.

**Figure 2:**
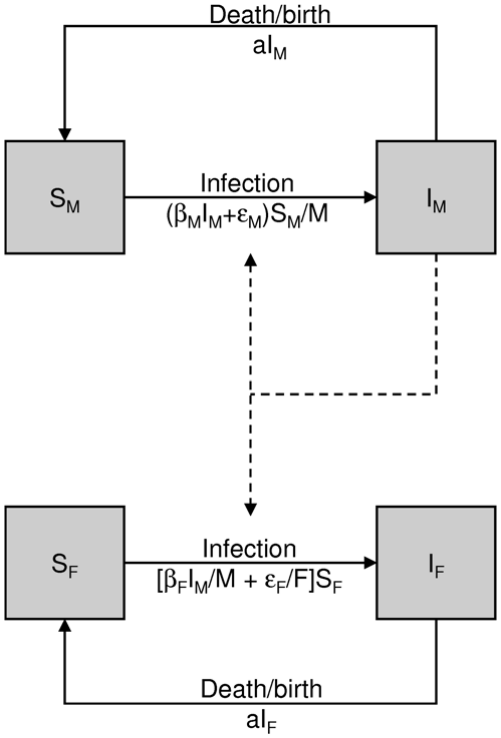
Compartmental representation of the model. The model includes both males (susceptible, *S_M_* or infected, *I_M_*) and females (susceptible, *S_F_* or infected, *I_F_*). Dashed arrows illustrate the fact that infected males are responsible for FIV transmission to both susceptible males and females.

Our mathematical model extends the classical Susceptible-Infected (*SI*) model (Fig. 2). We assume that all individuals of each population are equivalent, apart from their sex, the effect of sex on FIV transmission being too significant to be ignored. Indeed, of the 250 males captured in the study, 58 were seropositive (23.2%) compared to 22 of 249 (8.8%) females, which is highly significant (*χ* ^2^ = 13.80, 1 df, *p*<10^−4^). Moreover, males and females play different roles in the transmission of FIV [8], [12]. Since females rarely bite, they can be considered as non-transmitting of the virus. The sexual structure of the model is simply represented by splitting classes *S* and *I* into two sub-classes, one for each sex.

The age of individuals is not considered in our model, even though it may affect their behavior and, thus, their risk of becoming infected [3], [8], [13]. Moreover, due to long FIV infection duration, an accumulation of infected cases develops in older age cohorts. To represent the effects of age in a simplified way, we assume that the mean age of cats in the population may act as a risk factor for FIV transmission. This is justified since, here, we mainly focus on the global prevalence of FIV within populations without reference to the age-distribution of infections.

We assume a proportionate mixing law for the incidence function of FIV between males, which is more appropriate in social species [22], [23]. Transmission between males of the same population occurs at a rate *β_M_/M*, where *M* is the total number of males in the population, and susceptible females are infected by infected males from their population at a rate *β_F_/M*. The constants *β_M_* and *β_F_* are proportional to the rate at which males are involved in fights and to the rate at which females mate, respectively. We assume constant numbers of males (*M*) and females (*F*) within each population, whereby dead cats are instantaneously replaced by newborn cats. Since vertical transmission is very unlikely in the field [7], [24]–[26], all newborns are classified as susceptible to infection. Infected cats die at a rate *a*. Susceptible cats also die, but since they are instantaneously replaced by susceptible (and thus equivalent) newborn cats, their death is not explicitly modeled.

For the sake of simplicity, we assume that populations are not explicitly connected, such that the numbers of infected cats in the different populations are independent random variables. To avoid the definitive extinction of the virus from the populations we assume regular infections from an external source, e.g. another population. Males and females are infected from external sources at a rate *ε_M_/M* and *ε_F_/F*, respectively. The rates *ε_M_* and *ε_F_* are termed the external transmission rates.

The model is based on a continuous-time Markov process. Since we consider independent populations and constant numbers of males and females, the following set of *(M+1)(F+1)* ordinary differential equations describes the model (see [27] for an example of demonstration of differential equations representing continuous-time Markovian processes).

For 0≤*m*≤*M* and 0≤*f*≤*F* we have:
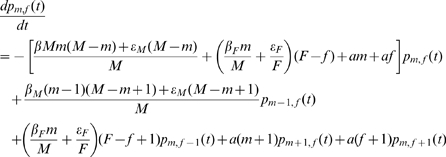



where *p_m,f_(t)* is the probability of having exactly *m* infected males and *f* infected females in the population at a time *t* (*I_M_ = m* and *I_F_ = f*). We fix *p_m,f_* ≡0 if *m* = −1, *m* = *M*+1, *f* = −1 or *f* = *F*+1.

In this model, the spread of FIV in males is independent of the number of infected females. As a result, the probability of finding exactly *m* infected males in the population (given by 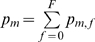) is independent of the female transmission rates (*β_F_* and *ε_F_*) and hence of the proportion of infected females in the population. The distribution of the number of infected males given by the model can also be compared with male infection prevalence data, independently of female infection prevalence. We define this model as the “male transmission model”. It is equivalent to a classical *SI* model [28].

#### 2.3. Influence of risk factors on the model parameters – Models *H^1^*

As discussed earlier, our purpose here is to measure the influence of some factors on the rate at which the virus spreads within or between populations. Two types of risk factors are tested here. The first ones concern the impact of demographic parameters (such as the number of cats within the population) on the virus transmission rate between cats of the same population. The second ones are not really risk factors. Behavioral observations suggest networks of connectivity between the different populations. The objective is to estimate whether introducing this information on the probability of disease reintroductions within populations produces significant predictive improvements, compared to models where external reintroduction rates are simply constants.

Firstly, we try to improve the goodness-of-fit of the observed data by assuming that both within-population transmission rates *β_M_* and *β_F_* depend on the **demographic characteristics** of the cat population:
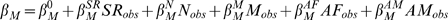


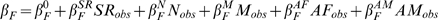



where *SR_obs_*, *N_obs_*, *M_obs_*, *AF_obs_* and *AM_obs_* are the observed values for the sex-ratio, the population size, the number of males in the population (*M_obs_* = *SR_obs_N_obs_*) and the mean age of captured males and females, respectively; considering these characteristics is intuitive since all of them may affect the social structure of the population and, hence, the transmission rates of FIV. 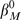, 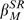, 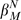, 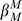, 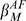, 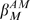, 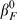, 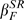, 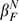, 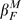, 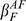 and 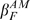 are the (linear) parameters that quantify the effects of these five demographic characteristics on the transmission rates *β_M_* and *β_F_*. Note that, *a priori*, the coefficients can have negative values and hence predict negative transmission rates. We fix a minimum value (10^−4^) below which *β_M_* and *β_F_* cannot fall since negative transmission rates are not allowed in the model. For the sake of simplicity, we assume that the external transmission rates *ε_M_* and *ε_F_* are not affected by the risk factors presented above.

We define as *H^0^* the model where 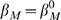 and 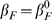, the four model parameters (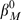, 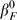, 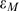 and 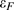) being positive. As a general definition, models involving other parameters are called *H(λ)*, where *λ* denotes the set of free (non-zero) parameters in the model that are not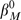, 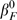, 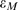 and 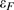.

Then we investigate the possibility that, all other parameters being equal, the external transmission rates (*ε_M_* and *ε_F_*) may differ between cat populations due to their **spatial organization**. Indeed, behavioral observations suggest a network of contacts between the different populations (see Fig. 1, solid arrows), which can be simplified by dividing the study area into three distinct metapopulations (see Fig. 1, grey areas). Since we do not model spatial structure explicitly, we assume that connectivity between populations affects external transmission rate. We define the resulting “neighboring” models and “metapopulation” models as follows.

A potential neighboring network has been suggested by behavioral observations (see Fig. 1). Intuitively, when there is a high FIV prevalence in males in neighboring populations, the external transmission rate of FIV should be higher. For this reason, we propose that the external transmission rate of FIV within a population could be considered as an affine function of the number of infected males in the neighboring populations (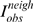):
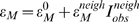


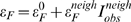



We refer to this model as the “neighboring model” *H_neigh_(λ)*, where *λ* denotes the set of free parameters in the model (in addition to 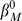, 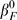, 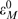 and 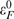 that are always freely variable).

The metapopulation model considers that viral exchange is more intense between populations from the same metapopulation than between populations from different metapopulations. A simple way to test this hypothesis is to assume that populations belonging to the same metapopulation have the same external transmission rate, and that this external transmission rate differs between populations from different metapopulations. We define the “male metapopulation model” 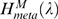, where 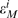 represents the value of 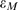 in metapopulation *i*, and *λ* denotes the set of free parameters in the model (in addition to to 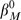, 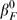, 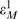, 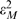 and 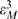 that are always freely variable). Note that in this model the only parameter that differs between cat populations is 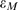, which varies among metapopulations (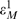, 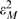, 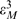 depending on the metapopulation).

Finally, we also define the “female metapopulation model” 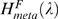, which is strictly equivalent to 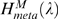, except that it pertains to female external transmission rates.

#### 2.4. Comparing models to data

Models cannot be directly compared with data because they predict distributions for the **total** number of infected and susceptible males and females in the population, whereas data are just **samples** of the real total numbers, *i.e*. the probability of capture is strictly below 1. To simplify, we assume that the total number of males and females in the populations are proportional to their observed values, i.e. *M = M_capt_(1+p_NC_)* and *F = F_capt_(1+p_NC_)*, where *p_NC_* is a constant (*1/(1+p_NC_)* is the proportion of captured cats) and *M* and *F* are the real numbers of males and females within the population, respectively. Based on the ratio between the number of cats captured through baited traps and the number of cats observed through intense monitoring in each population, we estimate that *p_NC_* is equal to 0.3 in average.

We assume that FIV is present in this area for a long period of time, corresponding to the stationary state of the distribution. So data are compared to this state. Note that the fact that the distribution is stationary does not mean that the population is at the equilibrium (i.e. endemic state), but only that epidemic, endemic and extinction events may succeed, and this being considered a population has a time-independent probability of being in each of its possible states.

Stationary distributions of the model, *i.e*. probabilities of finding exactly *m* infected males (for all 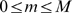) and *f* infected females (for all 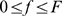) in the population, generate a distribution of possible outcomes *d_0_* for the total number of cats. To incorporate the fact that data are missing for non-captured individuals, we add a hypergeometric sampling element to the distribution *d_0_* (in other words data are the result of a random sampling of the entire population). This leads to the distribution *d* to which data can be compared [29]: 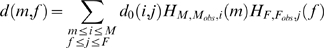
where *H_x,y,z_* is the hyper-geometric law of integer parameters *x*, *y* and *z*, which is defined when *max(y,z) ≤x* for all integers *t* satisfying *t ≤min(x,y)* and *z−t ≤x−y* by:
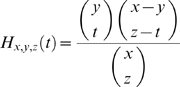



The distribution *d* is then equal to the distribution *d_0_* after sampling a proportion *1/(1+p_NC_)* of the population. In other words, *d_0_* is the asymptotic distribution of the number of infected males and females, after sampling a proportion *1/(1+p_NC_)* of the population.

#### 2.5. Model selection

Each of the models presented above can be summarized by the set of parameters that may vary freely – other parameters being fixed. Let us consider a model *H*. For each value *θ* of the free parameters in the freely variable parameter space *Θ* (*θ* is a vector of the values of all the free parameters), we can calculate for each population *k* the probability of generating the number of infected males and females actually observed. We call it *L_k_*(*θ|D_k_*), where *D_k_* represents the data restricted to population *k*; *D_k_* is defined by the number of infected males and females in population *k*.

Since we assumed that populations are independent, we can easily calculate the likelihood of the data *D* with the model *H*:
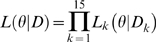



Now, if we consider two models *H^1^* and *H^2^*, the two models are compared using the maximum likelihood ratio statistics defined by:
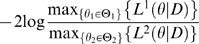



We use the classical approximation that, under regularity conditions, the likelihood ratio follows a *χ*-square distribution with *r* degrees of freedom, where *r* is the difference in the number of free parameters between models *H^2^* and *H^1^*.

#### 2.6. Determining confidence intervals for the parameters

Another important objective of mathematical modeling is to calibrate the selected model, *i.e*. the model selected from the previously described process (see Section 2.4 above) by determining confidence intervals for its parameters. We consider a model *H* with a given set of freely varying parameters that defines a vector (*θ*); *i.e*. each component of *θ* is a parameter of the model. Within each population, the model predicts a distribution for the number of infected cats (male or female). Each possible model outcome (defined as a vector of 30 integers representing the number of infected males and females within each of the 15 populations) has a probability of occurrence. What we want to determine is the values *θ* of the free parameters for which the observed data is a plausible outcome of the model. We accept that the data is a plausible outcome of the model when its likelihood is within the range of likelihood values of typical model outcomes, as described below.

For each vector *θ*, we determine the threshold *L_0.05_(θ)* such that 95% of the model outcomes have a likelihood value larger than *L_0.05_(θ)*. We now look at the likelihood of the observed data under the model parameters, defined above as *L(θ|D)*. Again we explore the parameter space. The confidence region *Θ_C_* can be defined as *Θ_C_* = {*θ∈Θ / L(θ|D)>L_0.05_(θ)*}. Thus, for *Θ_C_* the observed data is a likely model outcome. Since the model often has several free parameters, then the 95% confidence interval is, in fact, a region of the multi-dimensional parameter space of the free parameters (*Θ*). For that reason we use the term “confidence region” rather than “confidence interval”.

Finally, note that in the models the only parameter value we fix *a priori* is the mortality rate of FIV infected individuals (*a*). Since the model is analyzed at equilibrium, changing the mortality rate of infected individuals only results in a change in time scale. To remain consistent with cat-FIV interaction characteristics, we fix *a* = 0.0208 month^−1^, so that infected cats have a 4-year life expectancy [8]. The model time unit is the month.

#### 2.7. Computational procedure

Computationnal procedures are performed with Matlab. Stationary distributions of FIV prevalence in males and females are obtained by resolving the linear system corresponding to *dp_m,f_/dt* = 0. Maximum of the likelihood function are computed using a conjugate gradient method.

## Results

### 1. Description of the data

The cat number, sex-ratio, mean age of the males and females and percentage of FIV positive males and females captured in each population are given in Table 1.

**Table 1:** Total number of sampled cats, adult sex-ratio, number of FIV seropositive individuals (FIV+) and mean age of captured males and females in each population.

Population	Cats sampled	No. Males (sex ratio)	FIV+ males	FIV+ females	FIV+ total	Mean age of males	Mean age of females
Allamps (All)	28	12 (0.43)	4 (0.33)	1 (0.06)	5 (0.18)	3.79	4.46
Barisey-au-Plain (BaP)	24	10 (0.42)	2 (0.20)	1 (0.07)	3 (0.13)	4.75	4.57
Barisey-la-Côte (BlC)	34	16 (0.47)	5 (0.31)	0 (0)	5 (0.15)	3.85	5.60
Brixey-aux-Chanoines (BaC)	15	10 (0.67)	3 (0.30)	1 (0.20)	4 (0.27)	2.56	3.08
Champougny (Cha)	26	15 (0.58)	5 (0.33)	2 (0.18)	7 (0.27)	1.54	2.33
Clerey-la-Côte (ClC)	13	7 (0.54)	1 (0.14)	1 (0.17)	2 (0.15)	2.69	3.06
Jubanville (Jub)	50	26 (0.52)	0 (0)	1 (0.04)	1 (0.02)	5.25	5.42
Maxey-sur-Meuse (MsM)	29	16 (0.55)	1 (0.06)	0 (0)	1 (0.03)	2.31	3.05
Mont-l'Etroit (MoE)	19	10 (0.53)	0 (0)	0 (0)	0 (0)	2.40	2.37
Pagny-la-Blanche-Côte (PBC)	61	27 (0.44)	4 (0.15)	2 (0.06)	6 (0.10)	2.17	2.83
Punerot (Pun)	42	19 (0.45)	8 (0.42)	3 (0.13)	11 (0.26)	2.11	2.79
Ruppes (Rup)	29	23 (0.79)	6 (0.26)	2 (0.33)	8 (0.28)	2.95	2.32
Sauvigny (Sau)	71	34 (0.48)	11 (0.32)	4 (0.11)	15 (0.21)	3.36	3.21
Traveron (Tra)	22	11 (0.50)	2 (0.18)	3 (0.27)	5 (0.23)	2.84	3.72
Vaunes-le-Chatel (VlC)	36	14 (0.39)	6 (0.43)	1 (0.05)	7 (0.19)	3.56	4.38
**Total**	499	250 (0.50)	58 (0.23)	22 (0.09)	80 (0.16)	3.08	3.55

A total of 499 cats were sampled and tested for FIV in the 15 populations. There was large variability in the number of cats sampled due to large differences in population sizes, ranging from 13 cats in Clerey-la-Côte to 71 in Sauvigny. The overall sex-ratio is close to 50% but with differences between populations, although it does not differ statistically from a 50∶50 binomial distribution (χ^2^ = 17.21, 15 df, *p* = 0.31). However, in Ruppes the sex-ratio is rather high (0.79) and this value significantly differs from 0.5 when applying a Bonferroni correction for multiple tests (*p*<0.05).

For each captured cat, we estimated its age following Pascal and Castanet [30], and then the mean age of males and females in each population. For the entire study area the mean age is 3.08 years for males and 3.55 years for females; ranging from 1.54 years in Champougny to 5.25 years in Jubainville for males and from 2.32 years in Ruppes to 5.60 years in Barisey-la-Côte for females. It is also interesting to note that there is a strong correlation between the mean age of males and females in the studied populations (*r* = 0.85).

Finally, as previously documented, the global prevalence of FIV differs greatly between sexes (23% in males compared to 9% in females), with an average FIV prevalence in the entire study area of approximately 16%. There is significant variability in FIV prevalence between populations, especially in males, where data show significant extra-Binomial dispersion (Fisher's exact test with simulated *p*-value: *p*≈0.006). In contrast, the variability in FIV prevalence in females observed between populations is in agreement with the expectations of a binomial distribution (Fisher's exact test with simulated *p*-value, *p*≈0.183).

### 2. Qualitative analysis of FIV-prevalence and persistence with the dynamical model

Here we perform a rapid analysis of the mathematical model, this type of model having been studied in more detail elsewhere [28], [31]. For the sake of simplicity, we focus on the real distribution of FIV prevalence in males (the results are thus independent of *β_F_*, *F* and *ε_F_*); we assume *p_NC_* = 0, *i.e*. all individuals of the population have been sampled.

**Figure 3:**
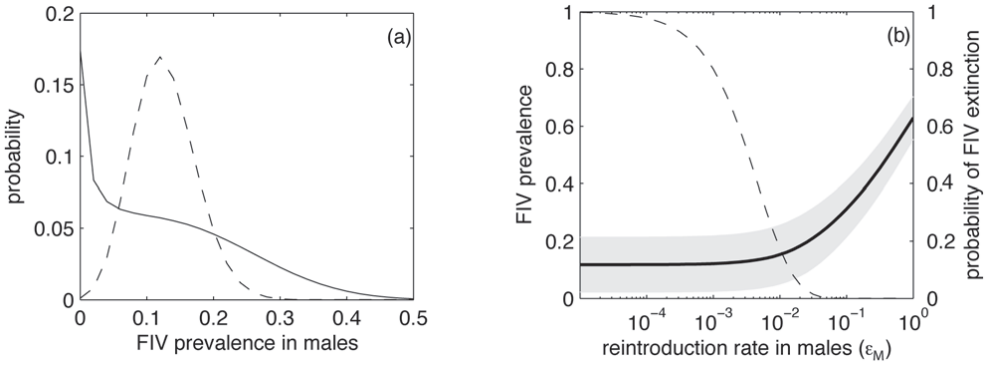
Results of the model. Parameter values: *β_M_* = 0.025 and *M* = 50. (a) Distribution of FIV prevalence in males (solid line, *ε_M_* = 0.01). A binomial distribution having the same mean is also represented (dashed line) and (b) the effect of the male external transmission rate (*ε_M_*) on mean FIV prevalence conditioned to non-extinction (solid line, left axis, the grey area represents the standard deviation of FIV prevalence) and on the probability of FIV extinction (dashed line, right axis).

First, we look at the distribution of FIV prevalence in males for arbitrarily fixed values of the parameters: *β_M_* = 0.025, *M* = 50 and *ε_M_* = 0.01 (Fig. 3a, solid line). For clarity, we plot the distribution of FIV prevalence as a continuous line, although the distribution is discrete. The probability of finding no infected cats in the population is high (17%). The mean prevalence of FIV is 12.66% and in 95% of the model outcomes the FIV prevalence ranges between 0 and 32%. This predicted distribution of FIV prevalence in males differs from a binomial one (a distribution frequently used in risk factor analysis, [8], [9]) having the same mean (Fig. 3a, dashed line). For a binomial distribution, the probability of finding no infected individuals in the population is much smaller (0.1%) and the confidence interval for FIV prevalence is [0.01; 0.20].

In Fig. 3b we analyze the effect of the external transmission rate on the mean and standard deviation of FIV prevalence in males. We focus on the distribution conditioned to non-extinction and, in parallel, we plot the probability of FIV extinction from the population (dashed line, right axis). Unsurprisingly, the probability of FIV extinction decreases with increasing external transmission rate (*ε_M_*). More interestingly, below a given threshold (here *ε_M_* = 10^−3^) the distribution of FIV prevalence is not affected by *ε_M_*, meaning that infrequent infections of FIV from external sources have almost no effect on FIV transmission within already infected populations. Under these circumstances, external infections only affect the frequency of extinction of the virus. Above the threshold, the mean prevalence of FIV increases with *ε_M_*. Thus, external infections are an important component of FIV transmission, even within already infected populations.

This result may have important implications. For example, in our data only two of the 15 populations have no infected males. This indicates that the external transmission rates of FIV within our populations must be large enough such that there are infected males in at least 13 out of the 15 populations. Under such external transmission rates, is the spread of FIV within already infected populations affected by external infections or is external infection only important for the long-term persistence of the virus? This question will be addressed later when we provide estimates for the parameters.

### 3. Analysis of the observed data using the dynamic model

#### 3.1. Effect of demographic risk factors

Now we consider the full model, including both males and females, and compare how integrating the different risk factors increases the goodness-of-fit to our observations using likelihood ratio tests. We performed the data analysis with each of the following values for the proportion of non-captured cats (*p_NC_*): 0.15, 0.30 and 0.45. Since the results obtained from these three values are very similar, we only show those obtained for *p_NC_* = 0.30. It is important to note that from now on, likelihoods are calculated with the distributions of FIV prevalence in males and females, without removing the cases of extinction (*i.e*. we use the distribution *d*). The probability of observing zero infected individuals is an important characteristic of the models, and removing extinction cases would lead to lose very important information, especially relating to the external virus transmission rate.

To start with, we look at the impact of the population characteristics (the sex-ratio in captured cats *SR_obs_*; the estimated population size, *i.e*. the number of captured cats *N_obs_*; the number of captured males in the cat population *M_obs_* = *SR_obs_ N_obs_*; and the mean age of captured males and females, *AM_obs_* and *AF_obs_*, respectively). Results are summarized in Table 2. The only significant effect we found is associated with the effect of mean age of males (χ^2^ = 4.09, 1 df, *p* = 0.043) and females (χ^2^ = 5.335, 1 df, *p* = 0.02) on the male-to-female transmission rate (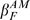 and 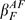, respectively).

**Table 2:** analysis of FIV risk factors.

Model	Value of 2 ln(L/L^0^)	*p*-value	df
**Social parameters**			
*H(* 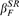 *)*	2.639	0.105	1
*H(* 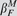 *)*	1.055	0.219	1
*H(* 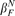 *)*	0.573	0.447	1
*H(* 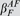 *)*	5.316	0.021	1
*H(* 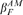 *)*	4.094	0.043	1
*H(* 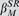 *)*	0.063	0.803	1
*H(* 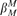 *)*	0.264	0.610	1
*H(* 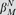 *)*	0.390	0.535	1
*H(* 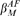 *)*	1.425	0.234	1
*H(* 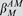 *)*	1.342	0.246	1
**Spatial parameters**			
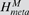	2.828	0.243	2
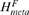	3.572	0.168	2
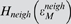	0.003	0.956	1
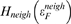	2.634	0.105	1

Interestingly, there is a negative correlation between the mean ages of males and females and FIV prevalence (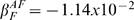
for model 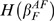 and 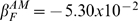 for model 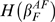).

This means that the effect of age on FIV prevalence observed here is not due to the accumulation of FIV cases with age. The *p* values are rather large (*p* = 0.02 and *p* = 0.043), especially considering the large number of tests performed. Unfortunately, we cannot apply a simple Bonferroni correction for multiple tests because of dependence among the different tests performed. Due to the strong correlation between the mean age of males and females, it is not surprising that both variables have the same significant effect on the male-to-female transmission rate. It seems more likely that only one of the two variables has a real biological effect, the effect of the other one being due to correlation. Due to the strong correlation between the two variables, we cannot rule out a role for mean age of males on the male-to-female transmission rate.

#### 3.2. Integrating the suggested spatial structures does not improve the model predictions

Now we compare the model *H^0^* with models where the external transmission rates are assumed to be different within each of the three cat metapopulations (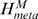 and 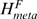, respectively) or where the external transmission rates of FIV in males and females are proportional to the prevalence of FIV in neighboring populations (models 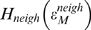 and 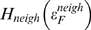, respectively). Under these circumstances, we find no significant improvement in the models compared to *H^0^* (see Table 2).

In summary, we found two potential risk factors for FIV: the mean ages of males and females that influence the FIV prevalence in females. These two factors are certainly linked because a large correlation exists between the two variables. Yet, considering the number of tests we performed and the relatively high *p* values we obtained, we cannot exclude the possibility that the simplest model *H^0^* alone explains the data. We found no risk factor for FIV spread between males. The maximum likelihood estimation of the parameters is *β_M_* = 2.71×10^−2^, *ε_M_* = 1.91×10^−2^, *β_F_* = 4.4×10^−3^ and *ε_F_* = 2.08×10^−2^ for model *H^0^*; *β_M_* = 2.66×10^−2^, *ε_M_* = 2.11×10^−2^, *β_F_* = 3.88×10^−2^ −1.14×10^−2^ *AF_obs_* and *ε_F_* = 1.92×10^−2^ for model 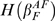 and *β_M_* = 2.70×10^−2^, *ε_M_* = 1.91×10^−2^, *β_F_* = 1.90×10^−2^ −5.30×10^−3^ *AM_obs_* and *ε_F_* = 2.30×10^−2^ for model 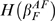.

### 4. Confidence intervals for the model parameters

#### 4.1. Male transmission parameters

Of all parameters, male transmission rates (*β_M_* and *ε_M_*) are the most important for understanding and controlling FIV transmission. Since we found no risk factors for the male transmission parameters, we investigate whether the male transmission model alone can reproduce the data for male FIV prevalence and, in particular, explain the large variability observed in the prevalence data. In other words, for what values of the parameters are the data a plausible outcome of the model? To answer this question, we look at the parameters for which the data falls within the 95% confidence intervals of the likelihoods of the model outcomes (see Material and Methods for more details).

**Figure 4:**
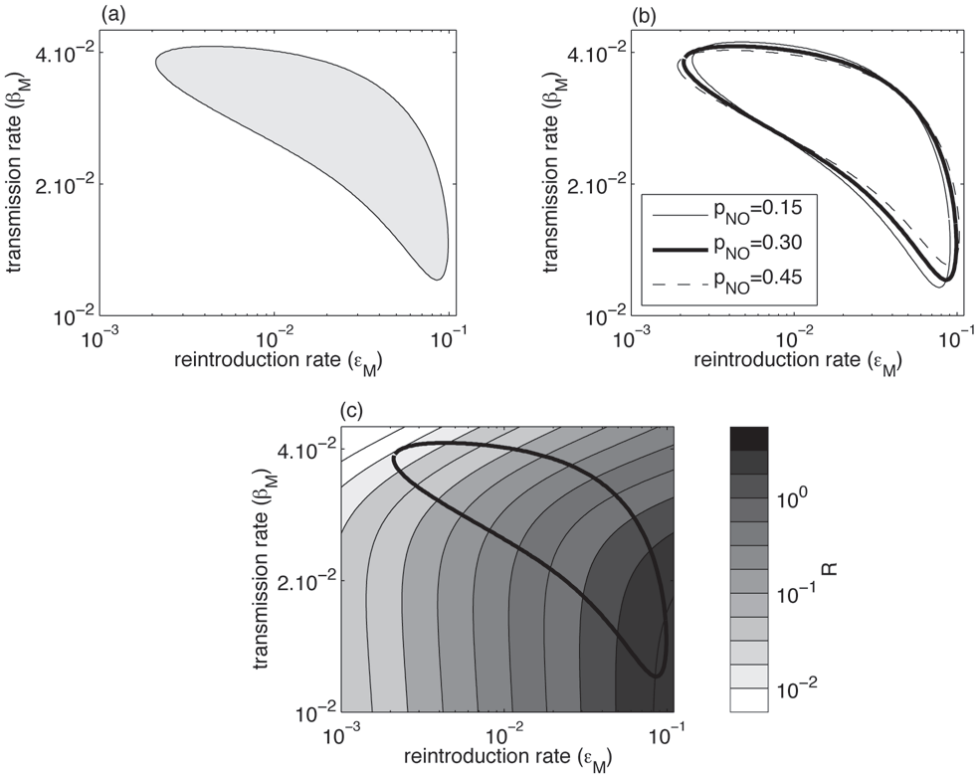
Confidence region of the (*β_M_, ε_M_*) space for the “male transmission model” parameters where the transmission coefficients are independent of risk factors. (a) For *p_NC_* = 0.30; (b) effect of *p_NC_* on the edge of the confidence region: *p_NC_* = 0.15 (solid thin line), *p_NC_* = 0.30 (bold solid line) and *p_NC_* = 0.45 (dashed thin line) and (c) value of the coefficient *R* (represented as the intensity of the grey-scaled color, see color bar on the right) in the confidence region (for *p_NC_* = 0.30).

We remove female prevalence data from the analysis because there is no female to male transmission of the virus. We determine the confidence region of the transmission rates *β_M_* and *ε_M,_* of the “male transmission model” parameter space for three different values of the proportion of non-captured cats: *p_NC_* = 0.30 (Fig. 4a, b), *p_NC_* = 0.15 and *p_NC_* = 0.45 (Fig. 4b). In Fig. 4b we superimpose these three confidence regions; only showing their boundaries. We conclude that *p_NC_* has a slight impact on the edge of the confidence region. If we project the region onto the *β_M_^0^* axis we obtain a 95% confidence interval for the male-to-male transmission rate *β_M_* ([1.30×10^−2^, 4.05×10^−2^]), with a maximum likelihood estimation of 2.68×10^−2^ for *p_NC_* = 0.30. Since the within-population basic reproductive number of FIV is given by *R_0_* = *β_M_/a*, we can derive that a 95% confidence interval for the estimation of the basic reproductive number is [0.626, 1.942], with a maximum likelihood estimation of 1.285, for *p_NC_* = 0.30. In a same manner we can estimate a confidence interval for *ε_M_*([1.93×10^−3^, 1.03×10^−1^]), with a maximum likelihood estimation of 2.03×10^−2^.

Finally we look at the impact of the external transmission rate *ε_M_* on FIV spread in already infected populations. We estimate the average size of a population as the mean number of observed males per population multiplied by 1+*p_NC_*, which is equal to 21 for *p_NC_* = 0.30. We divide the mean number of infected hosts calculated with the model for a population of average size conditioned to FIV non-extinction by the value obtained with the same parameters, but with an external transmission rate a hundred times lower. We denote *R* as this value minus one 
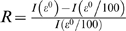
, where *I* is the mean number of infected individuals in the population. *R* is a proxy of the impact of external infections on FIV transmission in already infected populations. If external infections have a small effect compared to the within-population transmissions, then *R* will be close to 0 (see Fig. 3c). In contrast, if external infections have an important effect compared to the within-population transmissions, then *R* will be quite larger than 0.

For *p_NC_* = 0.30 we calculate *R* in a square region of the male transmission rate *(β_M_* and *ε_ M_)* parameter space (Fig. 4) and we superimpose on the same graph the edge of the confidence region. We observe that in the upper left corner of the parameter space (Fig. 4c) *R* is around 0.02, which means that at low external transmission rates, external infections only increase by 2% the prevalence of FIV and, so, have a very limited impact on the spread of FIV in already infected populations. In contrast, in the lower right corner of the confidence region *R* is around 2.5, which means that frequent external infections greatly increase FIV prevalence even in already infected populations.

#### 4.2. Confidence intervals for parameters influencing FIV prevalence in females

Now we investigate for which parameters values in the model including both males and females data are a plausible outcome of the model. We focus on the simplest model. First, it is interesting to know if, and for which set of parameters, the simplest model can fit the data. Second, since the effect of the mean age of populations is not highly significant, we do not believe it makes biological sense to take this factor into account here.

**Figure 5:**
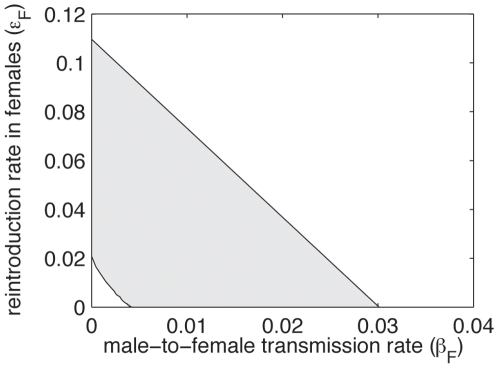
Projection of the confidence region of the model *H^0^* (including both males and females) on the parameter (*β_F_*, *ε_F_*) space for *p_NC_* = 0.30. The plotted region represents all paired values (*β_F_*,*ε_F_*) for which there exist concomitant values of the parameters *β_M_* and *ε_M,_* such that the observed data are a plausible outcome of the model.

Here, the parameter space is four-dimensional, so we cannot plot the confidence region. Since we are interested in determining the parameters directly influencing FIV prevalence in females, we simply plot the projection of the confidence region in the female transmission rate (*β_F_*, *ε_F_*) parameter space (Fig. 5). Fig. 5 thus shows all paired values of *β_F_* and *ε_F_* for which there exists concomitant values of the parameters *β_M_* and *ε_M,_* such that the observed data are a plausible outcome of the model.

Fig. 5 shows that there is an important dependency between *β_F_* and *ε_F_*. Increasing the value of *ε_F_* increases the mean prevalence in females and so the parameter *β_F_* must be decreased in order to explain the observed data. As a first approximation, the confidence region can be characterized by the relationship 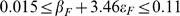.

Interestingly, the confidence region crosses the X and Y axis (see Fig. 5). This means that even if one of the two rates (*β_F_* or *ε_F_*) equals zero, then the model can still explain the data. In other words, the data may be explained by considering only infection of females by males of the same population, without external infections or, conversely, by only considering infections by males from other populations without within-population male-to-female infections. Overall, we cannot determine which source of infection for females (internal or external) is the most important in our study area.

### 5. Consistency of the male transmission model with FIV prevalence data in males

**Figure 6:**
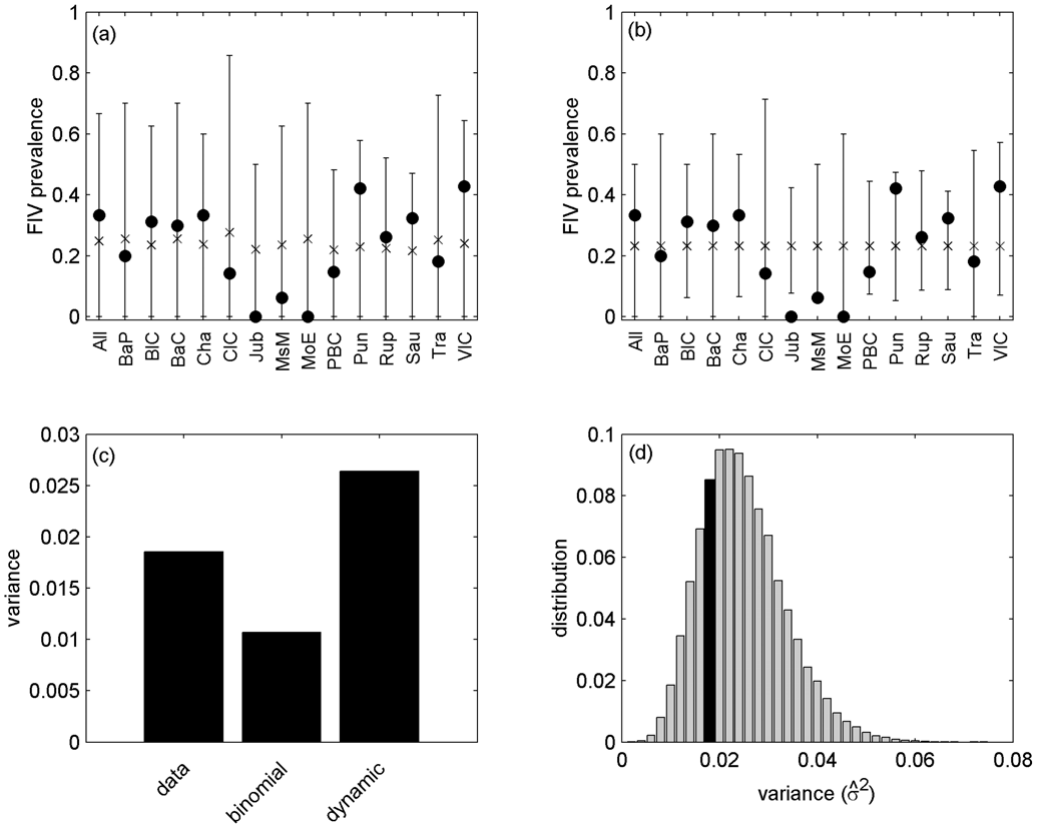
Consistency of the different models with male prevalence data. 95% confidence interval of FIV prevalence in the 15 population: with the dynamic epidemic model (a) and the binomial model (b). Crosses represent the mean of the distribution and black points the observed FIV prevalence data. (c) Comparison of the variance of male FIV prevalence between the 15 populations estimated from the field, and predicted by the binomial model and by the dynamic model. (d) Theoretical distribution of the estimated variance of FIV prevalence in males with the male transmission model. Black bar represents the observed value of this quantity.

In the previous section we have seen that data are a plausible outcome of the simple model for a large region of the parameters. In the present section we show how the simple male-transmission model (where the transmission rate is independent of risk factors) with maximum likelihood estimation of the parameters (*β_M_* = 2.68×10^−2^ and *ε_M_* = 2.03×10^−2^, here *p_NC_* = 0.30) fits to male prevalence data (Fig. 6a). For comparison we show the same graph using a binomial model (assuming independence between individuals regarding FIV, Fig. 6b).

As seen previously, the dynamic model predicts a very large variability of FIV prevalence in males within population (see Fig. 6a), which is larger than with the binomial model (see Fig. 6b). As a result observed FIV prevalence in males is always in the 95% confident region for the dynamic model (see Fig. 6a), but not for the binomial model (see Fig. 6b).

In Fig. 6c we show the variance predicted by the different models (with maximum likelihood estimations of their parameters) and we compare it with that estimated from the data (using 
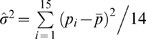
, where *p_i_* is the prevalence of FIV in males in population *i* and 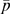 is the mean FIV prevalence). Again results show that the binomial model predicts a smaller variance than what is observed in the field (FIV prevalence data in males show around 73% more variance than what is expected by the binomial model), whereas the dynamic model shows an overestimated variance compared to what is estimated from data (but only around 42% larger).

To investigate whether the variance estimated from data is consistent with predictions of the dynamic model, we study the distribution of FIV prevalence in males expected by the male transmission model with maximum likelihood estimation of the parameter. In each population we simulate a male FIV prevalence according to the distribution *d* and then we estimate the variance in FIV prevalence in males between the 15 populations. We run 10,000 replicates and obtain a theoretical distribution of the estimated variance of FIV in males (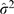, Fig. 6d). We find that the observed value of 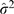 (black bar) is in fact a plausible outcome of with the dynamic model.

## Discussion

The spread of a transmissible disease in a host population is a dynamic process where the probability of individuals becoming infected depends on the number of infected individuals in their neighborhood. Nowadays, dynamic models of epidemics are widely accepted as efficient tools to help understand the spread and management of infectious diseases (see *e.g*. [32]–[35]). So it is not surprising that stochastic versions of these models have emerged during the past decade as the best way to analyze infectious diseases data (see *e.g*. [1], [14]–[19], [20], [36], [37]). Methods based on the comparison of stochastic epidemic models to data hence constitute natural tools to estimate how different factors may affect the spread and impact of infectious diseases.

### 1. Risk factors associated with FIV

Our dataset exhibits large variability in FIV prevalence in both males and females among populations. However, a rapid study of the dynamic model shows that, in such a model, great variability in FIV prevalence may be expected. The rate at which susceptible individuals become infected depends on the proportion of infected individuals in a population. If, by chance, the proportion of infected individuals becomes large then the number of new infections will increase, maintaining high infection prevalence for the next generation. By contrast, a low proportion of infected individuals decreases the number of infections in subsequent generations.

To investigate the possibility that the cats density, the sex-ratio, the number of males or the mean age of cats within the population may act as risk factors influencing the disease transmission rate, we performed a statistical analysis of the data using the sexually-structured *SI* model. Which population characteristics correlate with large FIV prevalence and so explain, in part, the variability in FIV prevalence? We found no such factors, except for mean ages. Interestingly, these ages have a negative effect on FIV prevalence in females, despite the accumulation of cases that occurs with age. One possible explanation is that the presence of older territorial males in some populations ensures greater social stability, which decreases the rate of at-risk (mating) contacts. Reversely, a negative correlation between FIV prevalence and age of cats could be due to the additional mortality induced by the virus. However, considering the weak impact of the infection on the life-expectancy of individuals, this explanation seems rather implausible to us.

In fact, these effects are not highly significant (*p* = 0.02 for the mean age of females and *p* = 0.043 for the mean age of males). Determining whether or not age affects the probability of becoming infected by FIV would require i) correction for the multiple tests performed and ii) correction for the effect of the accumulation of cases with age. Since this is beyond the scope of the work presented here, we cannot make definite conclusions on the effect of age.

### 2. Impact of external infections on FIV local prevalence

The cat populations observed in this study are of small size, and certainly are not large enough to retain the virus over long periods of time. Since we detected infected cats in 14 out of the 15 populations (and infected males in 13 of them), we can assume regular viral exchange between populations. Previous theoretical studies have shown the importance of the spatial dispersal of the FIV virus between populations [38]. Due to the topographic isolation of our study area, it seems reasonable to assert that viral exchange between the studied populations is primarily responsible for the reintroduction of the virus into populations where it has become extinct. We proposed two different virus dispersal networks between the populations, but neither significantly improved the goodness-of-fit to the observed data. Although our observations are most likely insufficient to capture the exact dispersal network between populations, the networks we analyzed should be quite realistic, because they are consistent with the natural barriers in the study area.

Lastly, it is important to note that a spatial correlation in FIV prevalence between connected populations can be observed only if external infections have a substantial impact on FIV prevalence within the population. An analysis of the confidence region of the male transmission parameters shows that the impact of external infections on FIV prevalence within populations is very limited for the smallest values of the external transmission rate (see Fig. 4c). In this case, the connectivity between populations cannot be revealed by a corresponding correlation in FIV prevalence. In contrast, for the highest values of external transmission rate in the confidence region, we can expect a correlation in FIV prevalence between connected populations. To sum up, the fact that no spatial correlation in FIV prevalence is observed may simply be due to the fact that external infections are relatively rare and thus play almost no role for disease prevalence in already infected populations.

### 3. About the approach

Logistic regression models are still widely used for the analysis of risk factors associated with infectious diseases, even though their over-simplified independence hypothesis is largely recognised as a limitation to their use [1], [14], [15]. The main difference between the two approaches, based on binomial and dynamic models, comes from the variability expected by their respective *H^0^* models, as illustrated in Fig. 3a. Binomial models predict much narrower distributions than dynamic models. The consequence is illustrated in Fig. 4, where we can see that the simple *SI* model accounts for the observed variability in FIV prevalence in males for a wide range of parameters. In contrast, the binomial test on the distribution of the infected cats among the 15 populations rejects the global binomial distribution hypothesis (*p*≈0.006). To explain the data with a logistic regression model that assumes binomial distributions, additional risk factors need to be invoked. With dynamic models, risk factors are not required to explain the variability in the male disease prevalence observed here. The implication of that is that bringing evidence for population risk factors in infectious disease requires large sample sizes. In our present case n = 499 is not large enough and further sampling is required to bring evidence of population risk factors for FIV transmission.

The model developed here is quite simple. In particular, it does not account for a potential difference in individuals’ infectivity between the acute and chronic phase of the infection. Such levels of complexity could be added to the method. This would make the model more realistic but also more complex, which was not our purpose here. The most important conclusion of the paper, i.e. that dynamic models predict much more variability than models where individuals are independent and hence are sufficient to explain highly variable prevalence data, would remain true for more complex model. Another model assumption is that we neglected the contacts with populations outside the study area (white rectangles in Fig. 1). Since we did not find an important effect of the number of infected neighbors on the disease prevalence in populations, we are confident that adding the neglected populations would not deeply affect our results.

### 4. Applications

The approach developed here is general and can easily extend to a wide variety of cat populations, but also to other host-parasite systems. It facilitates selection of the best model to describe data, which can be calibrated by determining confidence regions for the model parameters. The model can be used, for example, to test virtual management plans and to look at the expected results in the entire confidence region. This should assist in predicting the success one might expect with different management strategies. In the case of FIV, this study could help to rationalize the use of potential future vaccines or castration campaigns to limit the spread of the virus between males.

In the case of FIV, the approach gives us a 95% confidence interval for the model parameters, in particular for the basic reproductive number *R_0_* ([0.626, 1.942]), with a maximum likelihood estimate of 1.285. This value appears rather low, meaning that virus transmission is rather rare at the level of the population. This is not surprising, since experimental results indicate that most of the virus present in the saliva is not infectious [39], suggesting a weak efficiency in disease transmission [7]. Given the high frequency of fights between males in such populations and the low rate at which males acquire the infection (around once every four years in a hypothetical scenario where all males are infected), our results are consistent with the concept of a low probability of virus transmission from bites [9].
